# Telomeres and Longevity: A Cause or an Effect?

**DOI:** 10.3390/ijms20133233

**Published:** 2019-07-01

**Authors:** Huda Adwan Shekhidem, Lital Sharvit, Eva Leman, Irena Manov, Asael Roichman, Susanne Holtze, Derek M. Huffman, Haim Y. Cohen, Thomas Bernd Hildebrandt, Imad Shams, Gil Atzmon

**Affiliations:** 1 Department of Human Biology, University of Haifa, Haifa 3498838, Israel; 2 Institute of Evolution and Department of Evolutionary and Environmental Biology, University of Haifa, Haifa 3498838, Israel; 3 Faculty of Life Sciences, Bar-Ilan University, Ramat-Gan 5290002, Israel; 4 Leibniz Institute for Zoo and Wildlife Research, D-10315 Berlin, Germany; 5 Departments of Molecular Pharmacology, Medicine, and the Institute for Aging Research, Albert Einstein College of Medicine, Bronx, NY 10461, USA; 6 Freie Universität Berlin, D-14195 Berlin, Germany

**Keywords:** telomere length, naked mole-rats, blind mole-rats (*Spalax*), telomeres, long-lived, age, longevity

## Abstract

Telomere dynamics have been found to be better predictors of survival and mortality than chronological age. Telomeres, the caps that protect the end of linear chromosomes, are known to shorten with age, inducing cell senescence and aging. Furthermore, differences in age-related telomere attrition were established between short-lived and long-lived organisms. However, whether telomere length is a “biological thermometer” that reflects the biological state at a certain point in life or a biomarker that can influence biological conditions, delay senescence and promote longevity is still an ongoing debate. We cross-sectionally tested telomere length in different tissues of two long-lived (naked mole-rat and *Spalax*) and two short-lived (rat and mice) species to tease out this enigma. While blood telomere length of the naked mole-rat (NMR) did not shorten with age but rather showed a mild elongation, telomere length in three tissues tested in the Spalax declined with age, just like in short-lived rodents. These findings in the NMR, suggest an age buffering mechanism, while in *Spalax* tissues the shortening of the telomeres are in spite of its extreme longevity traits. Therefore, using long-lived species as models for understanding the role of telomeres in longevity is of great importance since they may encompass mechanisms that postpone aging.

## 1. Introduction

In the past decade, various studies on a wide range of animal models were conducted in the search for understanding the connection between telomere dynamics and longevity [1,2,3,4]. Telomeres are nucleoprotein structures that define the ends of linear chromosomes. They are composed of a repetitive DNA sequence, usually the conserved microsatellite TTAGGG in vertebrates, bound by a specialized six-protein complex known as “Shelterin”. Together they form a dynamic unit that protects the end of chromosomes from being recognized as broken DNA, and consequently prevents DNA end-joining, recombination or DNA repair that might lead to unstable chromosomes [5] and cell senescence. The telomere sequence is similar among all vertebrates so far investigated, indicating an ancient, conserved and highly effective system of genome protection [6]. Yet, the length of the repeated element, and hence telomere length, differs among chromosomes and individuals of the same species, as well as between species [7]. For instance, in humans, telomere length is of the order of 0.5–15 kilobase pairs while in rodents, reported telomere lengths vary from 10 to 72 kilobases [8]. Much less is known about telomere length outside model organisms but in recent years vast progress has been made in this field and data are starting to accumulate.

It is well established that in normal somatic cells, DNA polymerases are unable to fully replicate the linear chromosomes due to what is known as the “end-replication problem” [9]. This phenomenon causes telomeres to become progressively shortened after each cell division. Shortening of telomeres is usually compensated by the enzyme telomerase, or other less common telomere lengthening mechanisms [10,11]. Yet, most somatic tissues and adult stem cells do not express enough telomerase to retain telomere length and to compensate for the accelerated telomere attrition associated with aging. Upon shortening below a critical length and/or alterations in the functionality of the telomere-binding proteins, cells lose vitally important genes and enter “replicative senescence” which may be followed by cell death [12]. The rate at which telomeres shorten differs between different chromosomes, tissues, species [13,14] and physiological states. It is also worth noting that the expected rate of loss that is due to cell division is much lower than that which is usually observed. For instance, the estimated loss in human cells is 10 base pairs per cell division and yet the observed loss is 50–200 base pairs [15]. Hence, telomere shortening can serve as an important biomarker (biological thermometer) for life history traits such as aging and longevity as it is probably influenced by various factors including genetics, epigenetics and environmental factors [16,17,18,19].

Most of the studies conducted so far have primarily used short-lived animals as models for aging, like mice [20]. Yet, it is difficult to translate these findings to long-lived species like humans. However, a better understanding of how telomere dynamics vary across nature could be achieved by comparative biology experiments using long-lived animals, which may also eventually enable a better understanding for how telomeres are related to aging in humans.

Since telomere dynamics were found to be better predictors of survival and mortality than chronological age in wild populations [1], many cross-sectional and longitudinal studies have been conducted on different organisms with variations in maximum life span investigating the relationship between chronologic age and telomere shortening [14,21]. Yet, some studies have reported a lack of telomere shortening with age or even an increase in telomere length in organisms with exceptional longevity [14,22,23,24]. Therefore, studying telomere dynamics in long-lived organisms is of particular importance since they may have developed mechanisms that actively postpone senescence and promote effective defenses against the deteriorating effects of aging processes. 

The naked mole-rat (*Hetercephallus glabers*/NMR) and the blind mole-rat (*Spalax ehrenbergi*) are both considered excellent models for studying aging [25,26]. They both exhibit extraordinary longevity with a maximum lifespan of approximately 30 years in NMRs (10 times longer than any other rodent of the same size) [27] and 20 years in captivity for *Spalax* [28]. They exhibit lifelong maintenance of superior anti-aging mechanisms leading to unchanged physiological functions [29,30] and negligible senescence [31]. Moreover, both of these mole-rats live in a presumably relatively stressful environment due to their subterranean lifestyle where they experience darkness, low oxygen and high carbon dioxide concentrations. Despite all these common features, NMRs and *Spalax* belong to different families; they are different in size and have different social lifestyles (Table 1).

In the current study, we aimed to investigate the relationship between telomere length and age in these extremely long-lived animals. We tested blood telomeres in NMRs and three different tissues in *Spalax* and compared each one with a short-lived animal of their size. We hypothesize that telomere dynamics differ between short-lived mice and rats and the long-lived subterranean mammal’s NMR and *Spalax*, and thereby provide a better understanding as to whether telomere length is a biomarker or active component of the extreme longevity phenotype.

## 2. Results

### 2.1. NMRs and Mice

**Figure 1:**
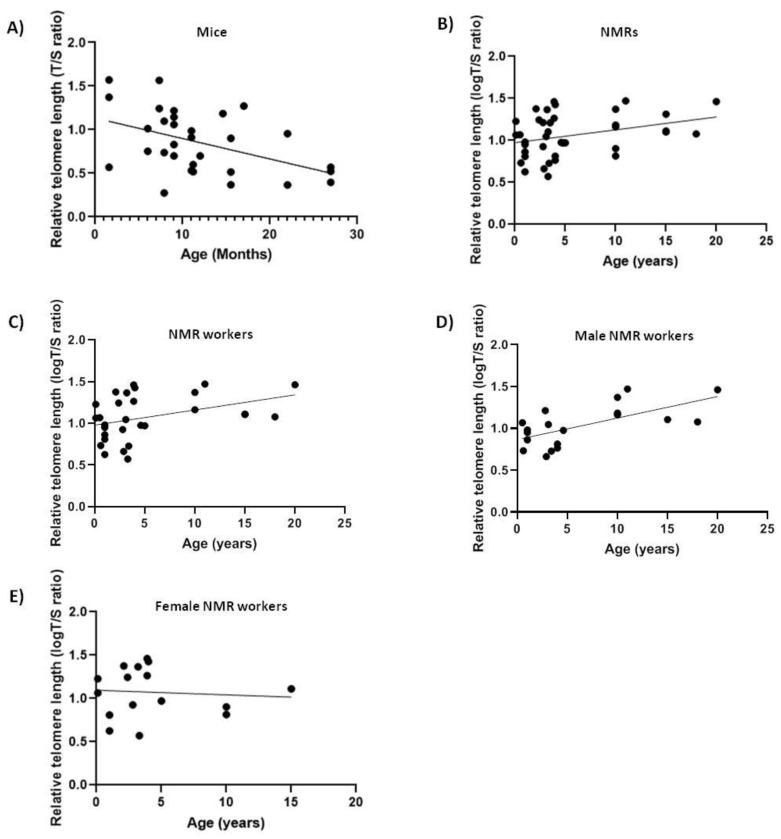
Relative telomere length (Telomere to Single copy gene (T/S) ratio) as a function of age in naked mole-rats (NMRs) and mice blood. The lines are the best-fit regressions through the data in (**A**) mice (slope = −0.02343, F_1,29_ = 7.741, *p* < 0.01, R^2^ = 0.2107); (**B**) NMRs (slope = +0.01538, F_1,38_ = 4.9691, *p* < 0.05, R^2^ = 0.1156); (**C**) NMR workers (slope = +0.01817, F_1,27_ = 4.672, *p* < 0.05, R^2^ = 0.1475); (**D**) male NMR workers (slope = +0.02571, F_1,17_ = 12.3, *p* < 0.05, R^2^ = 0.4197); (**E**) female NMR workers (not significant (ns)).

We analyzed relative telomere length (rTL) changes with age in blood DNA of 40 naked mole-rats and 31 mice in order to see if it differs between short-lived and long-lived animals. As expected, rTL decreased with age in mice (slope = −0.02343, F_1,29_ = 7.741, *p* < 0.01, R = −0.459) (Figure 1A) [32]. Surprisingly, telomere length did not decrease with age in NMRs (Figure 1B), but rather showed a slight increase (slope = +0.01538, F_1,38_ = 4.9691, *p* < 0.05, R = 0.34). Lack of telomere shortening with age in NMRs might suggest that protection of the telomeric sequence length is associated with the extraordinary longevity of this rodent and therefore an active component in the aging process. Furthermore, since our samples contained both male and female NMRs with different social status (queens, pashas and workers), which are known to behave differently, we next excluded the queens and pashas from our data and retested for correlation between telomere length and age (Figure 1C). Nevertheless, our results continued to show a positive mild correlation with age (Table 2) (slope = +0.01817, F_1,27_ = 4.672, *p* < 0.05, R = 0.384). 

Many studies have reported differences in longevity and in telomere dynamics between males and females [13]. Thus, we next tested the correlations between telomere length and age in males and females separately and found that only male workers showed a significant positive correlation (Table 2) between telomere length and age (slope = +0.02571, F_1,17_ = 12.3, *p* < 0.05, R = 0.6843) (Figure 1D), whilst females showed a non-significant positive correlation with age which we attribute to the small sample size (Figure 1E). 

### 2.2. Spalax and Rats

**Figure 2:**
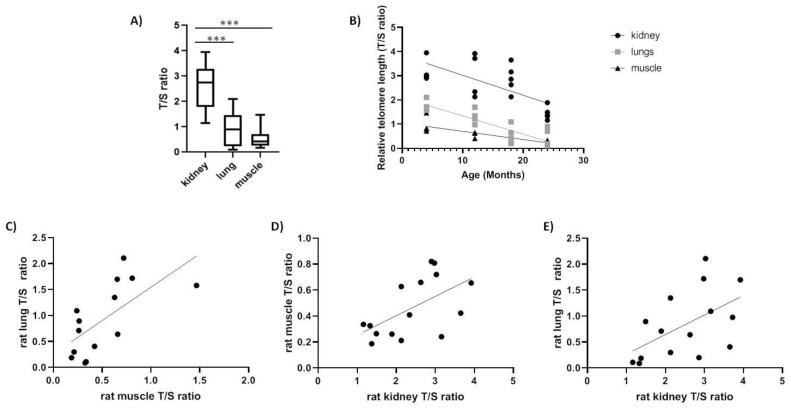
Relative telomere length (T/S ratio) in rat tissues. (**A**) range of relative telomere length (rTL) between all tissues (*N* = 16, *p* < 0.0001); (**B**) rTL as a function of age in kidney (slope = −0.08224, F_1,16_ = 13.78, *p* < 0.01, R^2^ = 0.4627), in lung (slope = −0.0752, F_1,15_ = 38.54, *p* < 0.0001, R^2^ = 0.7199) and in muscle (slope = −0.03385, F_1,14_ = 26.37, *p* < 0.01, R^2^ = 0.6532); (**C**) correlation between lung and muscle telomeres (*p* = 0.0395, r = 0.659); (**D**) correlation between kidney and muscle telomeres (*p* = 0.0278, r = 0.566); (**E**) correlation between kidney and lung telomeres (*p* = 0.0142, r = 0.535).

We tested rTL changes with age in kidney, muscle and lung tissues of a long-lived species (e.g., *Spalax*) and short-lived species (e.g., rats). In rats, the rTL was significantly higher in kidney compared with lung and muscle (*p* < 0.0001) in accordance with previous reports [13] and the shortest telomere length was detected in muscles (Figure 2A). Moreover, as expected, we found a decline in telomere length with age in all three tissues tested; kidney (slope = −0.08224, F_1,16_ = 13.78, *p* < 0.01, R = −0.68), lung (slope = −0.0752, F_1,15_ = 38.54, *p* < 0.0001, R = −0.808) and muscle (slope = −0.03385, F_1,14_ = 26.37, *p* < 0.01, R = −0.848) (Figure 2B). Furthermore, we found a significant positive correlation (Table 3) between tissues (kidney–lung: slope = +0.3763, F_1,13_ = 5.235, *p* < 0.05, R = 0.535; muscle–kidney: slope = +0.1490, F_1,13_ = 6.132, *p* < 0.05, R = 0.566; lung–muscle: slope = 1.287, F_1,12_ = 9.217, *p* < 0.05, R = 0.659) (Figure 2C–E).

**Figure 3:**
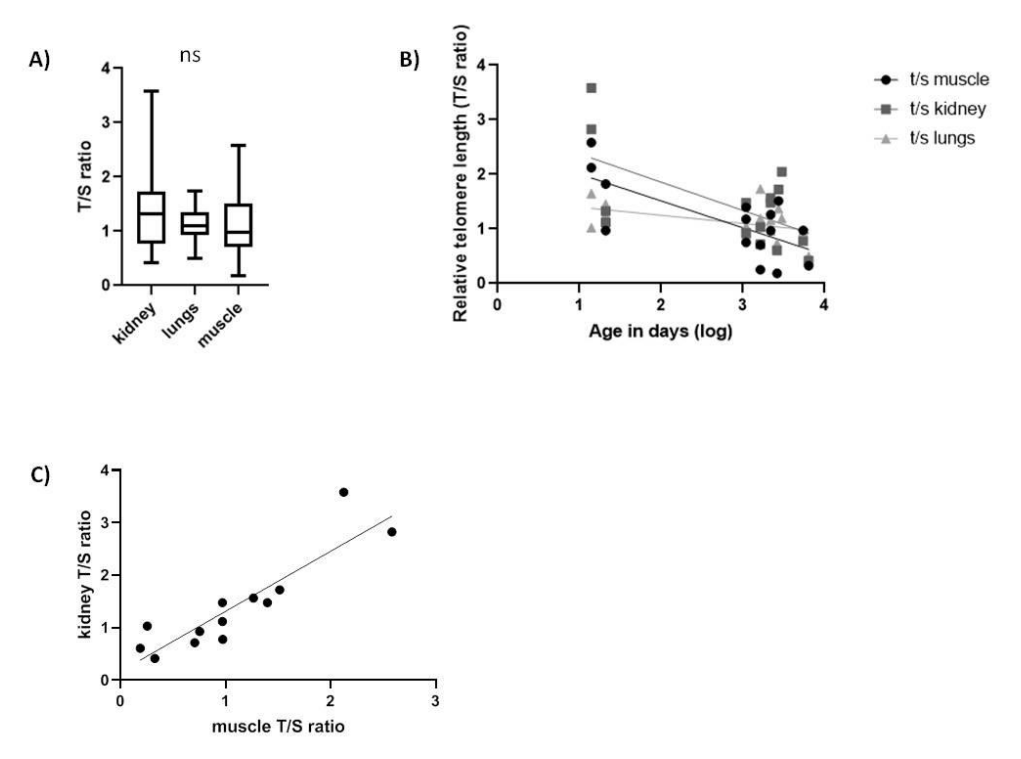
Relative telomere length (T/S ratio) in *Spalax* tissues. (**A**) range of rTL between all tissues (*N* = 15, ns); (**B**) rTL as a function of age in kidney (slope = −0.08451, F_1,13_ = 4.737, *p* < 0.05, R^2^ = 0.2671), in lung (slope = −0.03415, F_1,14_ = 5.759, *p* < 0.05, R^2^ = 0.2915) and in muscle (slope = −0.0706, F_1,13_ = 5.315, *p* < 0.05, R^2^ = 0.2902); (**C**) correlation between kidney and muscle telomeres (*p* = 0.001, r = 0.894).

In *Spalax*, the rTL was not significantly different among tissues (Figure 3A). Interestingly, we found a significant negative linear correlation between age and rTL in all three tissues tested (Figure 3B), similar to short-lived species. Subjects a few days old showed a high variability in the rTL in all tissues that declined with age faster in muscle (slope = −0.0706, F_1,13_ = 5.315, *p* < 0.05, R = −0.538) and kidney (slope = −0.08451, F_1,13_ = 4.737, *p* < 0.05, R = −0.5168) than in lung tissues (slope = −0.03415, F_1,14_ = 5.759, *p* < 0.05, R = −0.5398). Upon testing correlation in rTL among tissues, we only found a significant positive correlation (Table 3) between muscle and kidney (slope = +1.144, F_1,11_ = 43.86, *p* < 0.001, R = 0.894) (Figure 3C). 

**Figure 4:**
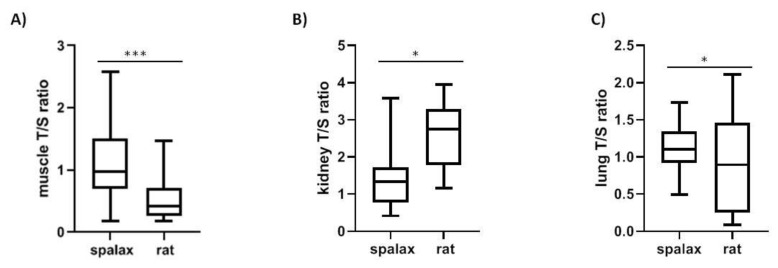
Telomere length in *Spalax* and rat in (**A**) muscle (*N* = 15, *p* < 0.0001); (**B**) kidney (*N* = 15, *p* < 0.05); (**C**) lung (*N* = 16, *p* < 0.05).

Telomere length in *Spalax* was longer in muscle and shorter in kidney compared to rats, (*p* < 0.01 muscle, *p* < 0.001 kidney) (Figure 4A,B). Furthermore, rTL differs significantly between *Spalax* and rat lungs (Figure 4C).

## 3. Discussion

Many studies have demonstrated that telomere length as well as the rate of telomere attrition can predict better life expectancy than chronological age [1,33,34]. Yet, most of these studies were conducted on various tissues of different short-lived animal models. Since it is difficult to translate these conclusions to long-lived species, such as humans, it is argued that long-lived animals may be better models for understanding the role of telomeres in the aging process. Telomere length in cross sectional studies have shown that short-lived animals face more rapid telomere shortening than long-lived ones [14]. Moreover, some of the studies on long-lived animals could not detect telomere shortening and hence it was proposed that long-lived animals possess more efficient mechanisms protecting against replicative senescence such as higher telomerase activity throughout life [35]. 

It is worth mentioning that recent studies have suggested that telomere-induced senescence may occur irrespective of the length of telomeres [12] and that the rate of increase in the percentage of short telomeres rather than the overall telomere length can predict lifespan [36]. Yet, in the case of NMRs and *Spalax*, it has already been established that, on average, they have long telomeres (Table 1) compared to humans and that these telomeres do not shorten in dying cultured cells [37,38], which lead us to assume that it is not the percentage of short telomeres but rather telomere length itself that is associated with their longevity. Furthermore, the epigenetic regulation of telomere length in aging, which we have previously reviewed in [39], is distinct from “epigenetic aging” and the epigenetic clock proposed by Horvath [40,41]. 

In this study, we cross-sectionally examined the average relative telomere length changes with age in two long-lived species; the NMR and the *Spalax*, while comparing each one to a short-lived species of comparable size (mice and rats, respectively). To our knowledge, this is the first attempt to study telomere length changes in these two species. We hypothesized that telomeres of long-lived animals may be active players in the aging process instead of mere biological markers in longevity determination, thereby contributing to their extraordinary longevity. 

The results of our study should be analyzed with regard to the tissues used. rTL is most commonly measured in blood cells (peripheral blood leukocytes) due to the ability to obtain multiple repeated samples using minimally-invasive sampling techniques. Most of the studies that addressed this topic showed positive correlations between blood telomere length and telomere length in other tissues, such as muscle and liver, which suggests that blood telomeres can be a suitable surrogate marker for telomere length in at least some tissues [42,43,44]. Our findings showed a decline in rTL in mice with age which is in accordance with previous studies conducted on mice telomeres [32]. However, rTL in NMR did not shorten but rather showed a mild, significant elongation with age. These results are similar to numerous other studies conducted on extremely long-lived animals like birds, trees, sand lizards and water python [14,22,23] that showed a positive relationship between telomere lengths and age. These findings can be further supported by another study that examined telomerase genes in NMRs and reported unique polymorphisms of the genes and promoter structure, which was suggested to have contributed to their slowed aging [45]. 

Moreover, one of the most common explanations for variability in telomere length among individuals of the same age is the acceleration of telomere loss by various stressors, such as oxidative stress [46]. Regardless of the fact that NMRs are subterranean rodents that experience a relatively stressful environment with low oxygen and high carbon dioxide concentrations, it is well established that they have high tolerance to hypoxia and hypercapnia and are resistant to damage by reactive oxygen species (ROS) [27] which might explain why NMR telomeres in blood did not shorten with age. 

Another unique life trait in NMRs is eusociality, where they live cooperatively in large colonies of up to 290 individuals with a single breeding female (queen) and one to three breeding males (pashas), while the rest of the individuals exhibit a division of labor. Therefore, when excluding pashas and queens from our data, a higher positive correlation between telomere length and age was observed and that is in accordance with the common assumption that queens and pashas exhibit different biology than their workers. 

Various studies have also demonstrated differences in telomeres between males and females [47], where it has been reported that females have longer telomeres than males, which is consistent with their increased longevity compared to males. Surprisingly, our results showed a positive correlation between telomere length and age only in males but not in females. One possible explanation is that female NMRs might rely on other mechanisms than telomerase for maintaining telomere length like recombination that occurs in long telomeres. Another possible explanation is the small female sample size used in this study [48].

Studies in rats have already established a decline in rTL with age in various tissues including lung and kidney [13]. These studies have also established that highly proliferative tissues have faster decline in telomere length with age than in minimally proliferative tissues such as muscle [49]. It is also well documented that, unlike humans, several rat somatic tissues express telomerase activity [38], but that does not always prevent telomere shortening. Our findings showed a decline in rTL with age in all rat tissues examined (kidney, lung and muscle), which is in accordance with what is already known in the literature. Here, we found significant differences in telomere length among tissues with longer telomeres reported in kidney with the sharpest decline, while telomeres in muscle were shorter and exhibited a slower decline. A possible explanation for this finding is that muscle telomeres in rats are short to begin with, and since myocytes rely on hypertrophy rather than proliferation, the decline with age was not as rapid. 

Similar to NMRs, *Spalax* also lead a subterranean lifestyle exposed to decreases in oxygen supplies which led them to develop physiological adaptations to their environment including tolerance to hypoxia. Despite these mechanisms, our findings showed that rTL decreased with age in kidney, muscle and lung of *Spalax* with a higher rate in muscle, which we attribute to the digging of borrows as well as their size and lifestyle. Notably, as we mentioned previously, despite the common characteristics shared between NMRs and *Spalax*, they belong to different families which might explain the differences in telomere dynamics. 

In summary, our results suggest that the NMR might have evolved age combating adaptations, such as telomere elongation in blood, by up-regulating telomerase. While similar to short-lived mice and rats, in tissues of long-lived *Spalax*, telomeres have deteriorated due to the rough environment. These findings may contribute to a better understanding of telomere-mediated aging processes and diseases in other important long-lived species like humans. Yet, further examinations of the NMR tissues and blood from *Spalax* as well as telomerase activity are needed in order to gain more knowledge about other fundamental questions in aging biology. 

## 4. Materials and Methods

### 4.1. Animals

We determined the relationship between relative telomere length and age in cross sectional samples from naked mole-rats (*Heterocephalus glaber*/NMRs), blind mole-rats (*Spalax*), male brown Norway (BN) rats (*Rattus norvegicus*) and mice (*Mus musculus*). Naked mole-rat blood samples were collected from a captive known-age colony maintained at the Leibniz-Institute for Zoo and Wildlife Research (Berlin, Germany) in artificial plexiglass labyrinths. Blind mole-rat samples (*Spalax*) were collected from open fields and were all kept under standard conditions in individual cages in the Animal Facility of the Institute of Evolution, University of Haifa. The exact age of *Spalax* cannot be determined since they do not breed in captivity, therefore, the stated age is an estimation of the minimal age (for adults, 2 y in the field is added to the time spent in captivity). *Spalax* tissues were harvested from animals euthanized by isoflurane overdose. *Spalax* protocol was approved by the Institutional Ethics Committee [Reference #316/14 (2014); 318/14 (2014); 420/16 (2016) and 580/18 (2018)]. Mice blood samples were attained from the facial vein of C57BL/6J females at the indicated ages. Mice were kept under specific pathogen free conditions, with free access to standard chow diet and water. Rat tissues were collected from male BN animals obtained from the National Institute of Aging (NIA) aged rodent colony (*n* = 5 per group) by the Einstein Nathan Shock Center Aging Physiology Core.

### 4.2. DNA Extraction

DNA was extracted from naked mole-rat and mice blood samples following the manufacturer’s protocol using a High Pure PCR Template Preparation Kit (Roche, Penzberg, Germany). Three tissues (lungs, muscles and kidneys) were dissected from *Spalax* and rats and immediately frozen and stored at −80 °C. Genomic DNA was extracted from these tissues using a High Pure PCR Template Preparation Kit (Roche, Germany). All DNA samples were tested for purity and integrity using a Nanophotometer NP80 (Implen, Munchen, Germany).

### 4.3. Telomere Measurement

The average relative telomere length was measured using qPCR (quantitative polymerase chain reaction) on a LightCycler 480 II (Roche, Germany) as described in Cawthon [50] and Axelrad [51] and modified for use in our four species animal models. The relative telomere length (T/S) of the samples was calculated as the ratio of the telomere concentration (T) to the single copy gene (S), relative to the reference sample standard curve.

Acidic ribosomal phosphoprotein P0 (36B4) gene was used as single copy gene for naked mole-rats and mice and Erythropoietin (EPO) gene was used as a single copy gene for *Spalax* and rats. A list of primer sequences can be found in the supplementary materials (Table S1). Both reactions for telomeres and single copy genes were run on the same plate in duplicate for each sample along with a negative control of water. All reactions used 10 ng of DNA in a final volume of 20 µL containing 10 µL SYBER green Master Mix, 2 µL primers, 6 µl water and 2 µL DNA sample. Reaction components and the LightCycler program are detailed in the supplementary materials (Tables S1 and S2).

In order to determine the efficiencies of each plate, samples were run against a standard curve produced by serially diluting a reference sample. The optimized concentrations for each species are listed in the supplementary materials (Table S3). All plate efficiencies were in the accepted range and only samples that fell within the bounds of the standard curve were further analyzed. 

### 4.4. Statistical Analysis

All statistical analyses were carried out in JMP12 (SAS Institute Inc., Cary, NC, USA) and GraphPad Prism8 (San Diego, CA, USA). Correlation between rTL and age was calculated using Pearson’s correlation test. The lines on the graphs represent simple linear regression adjusted to age. Statistical comparisons were made using the unpaired Student’s t-test for two groups and one-way analysis of variance (ANOVA) for multiple groups.

## Figures and Tables

**Table 1:** List of species included in the study.

Species	MLSP * (Years)	Body Size (g)	DNA Source	Average Telomere Length (kb)	Age Range	Number of Samples
Naked Mole-Rat (NMR)	31	40	EDTA whole blood	34	0–20 years	40
Mice	4	30	EDTA whole blood	72	0–27 months	31
Blind Mole-Rat (*Spalax*)	20.2	120	Kidney, lung, muscle	50	0–17.5 years	18
Rats	5	400	Kidney, lung, muscle	60	4–24 months	20

* Maximum Life Span.

**Table 2:** Telomere length correlation with age.

Animal	DNA Source	Correlation Coefficient
NMRs	Blood	0.334 *
	Workers blood	0.384 *
	Female blood	0.1096 ns
	Male blood	0.6843 **
Mice	Blood	−0.459 **
*Spalax*	Kidney	−0.5168 *
	Muscle	−0.538 *
	Lung	−0.5398 *
Rats	Kidney	−0.68 **
	Muscle	−0.848 ***
	Lung	−0.808 ***

* *p* < 0.05, ** *p* < 0.01 and *** *p* < 0.001.

**Table 3:** Telomere length correlation among tissues.

		Kidney	Muscle
*Spalax*	Kidney		0.894 ***
	Lung	0.1739 ns	0.306 ns
Rat	Kidney		0.566 *
	Lung	0.535 *	0.659 *

* *p* < 0.05, ** *p* < 0.01 and *** *p* < 0.001.

## Supplementary Materials

Supplementary materials can be found at https://www.mdpi.com/1422-0067/20/13/3233/s1.

## Abbreviations

NMR	Naked mole-rat
rTL	Relative telomere length
ns	Not significant
MLSP	Maximum lifespan
