# Supplementary material for: Telomeres and Longevity: A Cause or an Effect?
Source: Int J Mol Sci. 2019 Jul 1;20(13):3233. doi: 10.3390/ijms20133233 (PMC6651551; doi:10.3390/ijms20133233)
Supplement: Supplementary file 1 [file ijms-20-03233-s001.pdf]

Supplementary materials

Table S1. List of primers

Primer	Forward sequence	Reverse sequence	Species
Telo	5'-CGGTTTGTTTGGGTTTGGGTTTGGGTTTGGGTTTGGGTT-3'	5'-GGCTTGCCTTACCCTTACCCTTACCCTTACCCTTACCCT-3'	All
36B4	5'-ACTGGTCTGGGACCTGAGAAG-3'	5'-TCAATGGTGCCTCTGGAGATT-3'	NMR
36B4	5'-ACTGGTCTAGGACCCGAGAAG-3'	5'-TCAATGGTGCCTCTGGAG ATT-3'	mice
EPO	5'- GGCTGTAGAGGTCTGGCAAGG-3'	5'-GGAATTGGCTAGCACAGCCT-3'	*Spalax*
EPO	5'-ACGCCTCATTTGCGACAGTC-3'	5'-GCCCATTGTGACATTTTCTGC-3'	Rat

Table S2. Reaction components

**Telo**	**Telo x1**	**SCG x1**
DDW	6 µl	6 µl
Primer F	0.2 µl	0.6 µl
Primer R	1.8 µl	1.4 µl
RXN mix	10 µl	10 µl
DNA or serial dilution	2 µl	2 µl

S1. PCR program

Initial Denaturation - 95°C for 10 min

Denaturation - 95°C for 10 sec

Annealing - 60°C for 5 sec

Synthesis - 72°C for 11 sec

× 50 (Denaturation, Annealing, Synthesis)

Table S3. Standard curve concentrations

species	Top conc. (telo)	Top conc. (SCG)	Dilution factor
NMR+mice	6.1	1.8	1.68
Spalax +rat	50	50	10
